# How the evolution of multicellularity set the stage for cancer

**DOI:** 10.1038/bjc.2017.398

**Published:** 2018-01-16

**Authors:** Anna S Trigos, Richard B Pearson, Anthony T Papenfuss, David L Goode

**Affiliations:** 1 Computational Cancer Biology Program, Peter MacCallum Cancer Centre, Melbourne, VIC 3000, Australia; 2 Sir Peter MacCallum Department of Oncology, The University of Melbourne, Parkville, VIC 3010, Australia; 3 Department of Biochemistry and Molecular Biology, The University of Melbourne, Parkville, VIC 3010, Australia; 4 Department of Biochemistry and Molecular Biology, Monash University, Clayton, VIC 3168, Australia; 5 Bioinformatics Division, The Walter & Eliza Hall Institute of Medical Research, Parkville, VIC 3052, Australia

**Keywords:** evolution, systems biology, networks, atavism, multicellularity, network medicine

## Abstract

Neoplastic growth and many of the hallmark properties of cancer are driven by the disruption of molecular networks established during the emergence of multicellularity. Regulatory pathways and molecules that evolved to impose regulatory constraints upon networks established in earlier unicellular organisms enabled greater communication and coordination between the diverse cell types required for multicellularity, but also created liabilities in the form of points of vulnerability in the network that when mutated or dysregulated facilitate the development of cancer. These factors are usually overlooked in genomic analyses of cancer, but understanding where vulnerabilities to cancer lie in the networks of multicellular species would provide important new insights into how core molecular processes and gene regulation change during tumourigenesis. We describe how the evolutionary origins of genes influence their roles in cancer, and how connections formed between unicellular and multicellular genes that act as key regulatory hubs for normal tissue homeostasis can also contribute to malignant transformation when disrupted. Tumours in general are characterised by increased dependence on unicellular processes for survival, and major dysregulation of the control structures imposed on these processes during the evolution of multicellularity. Mounting molecular evidence suggests altered interactions at the interface between unicellular and multicellular genes play key roles in the initiation and progression of cancer. Furthermore, unicellular network regions activated in cancer show high degrees of robustness and plasticity, conferring increased adaptability to tumour cells by supporting effective responses to environmental pressures such as drug exposure. Examining how the links between multicellular and unicellular regions get disrupted in tumours has great potential to identify novel drivers of cancer, and to guide improvements to cancer treatment by identifying more effective therapeutic strategies. Recent successes in targeting unicellular processes by novel compounds underscore the logic of such approaches. Further gains could come from identifying genes at the interface between unicellular and multicellular processes and manipulating the communication between network regions of different evolutionary ages.

The hallmarks of cancer (Hanahan and Weinberg, 2011) provide an important unifying framework for studies of the molecular drivers of cancer. Distilling a set of common cellular and biochemical phenotypes shared by tumours from different tissues of origin and with unique sets of driver mutations, they articulate core principles of tumourigenesis and formed the basis for recent breakthroughs in cancer therapy. However, the guiding principles behind the establishment of these hallmark properties remain obscure, with little concordance with specific recurrent mutations.

The strong convergence towards common hallmark phenotypes is a reflection of the network structure of gene–gene and protein–protein interactions in human cells and how they dictate which genetic or epigenetic alterations are viable and beneficial during carcinogenesis. This network structure is the result of the action of selective pressures on gene and interaction innovations and alterations that have occurred during the evolutionary history of life. The evolution of multicellular life from unicellular ancestors, in particular, had major effects on the structure of these networks and has a major bearing on many cellular phenotypes and genetic alterations observed in cancer. A better understanding of these associations would aid in the discovery of new system-level properties of cancer, explain why specific alterations are preferred in cancer cells, and provide a gene prioritisation strategy to identify potential drug targets.

Consortia such as The Cancer Genome Atlas have released large amounts of high-quality genomics data for a variety of cancers, and many signalling, gene regulatory and protein–protein interaction networks of considerable size have become publicly available in the last decade (e.g., PathwayCommons; Cerami *et al*, 2011). These have allowed the emergence of new fields of research, such as network medicine (Barabasi *et al*, 2011), which have been successful in the development of promising treatment strategies based on network rewiring. Although the study of tumour development, progression and clonality has benefited from the introduction of evolutionary concepts, an understanding of these processes that accounts for the evolutionary history of cellular networks is lacking.

We propose that the shaping of gene regulatory networks during evolution, particularly the evolution of multicellularity from unicellular ancestors, led to unicellular and multicellular genes playing differing roles in cancer and created links within the network structure that are vulnerable to disruption that allow the development of cancer. Many of these vulnerabilities have been tackled clinically after being identified mechanistically, but we argue that incorporating evolutionary ages into system-level analysis can accelerate discovery of key vulnerabilities and pinpoint genes of interest, thus shedding light on new therapeutic strategies and aiding the discovery of drug targets, and streamlining the development of therapeutic strategies from a systems perspective. This was previously quite difficult due to technological limitations, but improvements in DNA sequencing have produced extensive and diverse new cancer genomics data sets and revealed the genome sequences of a growing array of species. Here we describe how a perspective on the evolutionary histories of the network of genes and proteins helps explain major tumour properties, including convergent evolution and drug resistance. We propose various strategies to incorporate the evolutionary histories of genes into systems-level analyses conducted in the context of network and Darwinian medicine (Greaves, 2007), to derive new candidate drug targets and develop treatment strategies that consider how the entire cellular network of genes and proteins has evolved over time.

## Signatures of macroevolutionary events on human molecular interaction networks underlie many properties of cancer

Interactions between genes and cellular processes in cells reflect major adaptations that occurred throughout evolutionary history. Multiple reports have associated past evolutionary events with current phenotypes, such as gene expression and essentiality with gene age (Kim *et al*, 2012; Popadin *et al*, 2014), suggesting latent encoding of phenotypic features within topological properties of networks. Processes such as the preferential formation of interactions between proteins that appeared during major evolutionary innovations (Chen *et al*, 2014), likely played important roles in defining basic common topological properties of networks in modern species, such as their number of interactions per gene (power-law distribution and scale-free topology) and their modular structure (Ravasz *et al*, 2002), which will have tangible and phenotypic consequences on the behaviour of molecular networks in present-day species, including humans.

**Figure 1:**
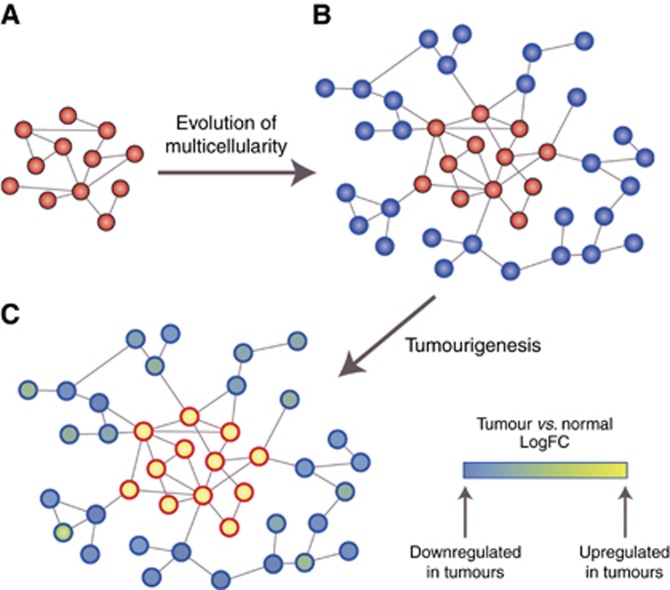
**The evolution of multicellularity led to network innovations with a particular signature during tumourigenesis.** (**A**) A simple representation of a molecular network of a hypothetical primitive unicellular organism, which would be compact with high connectivity between genes (red). (**B**) In a multicellular descendent of the species whose network was represented in (**A**), genes supporting multicellular functions (blue) (multicellular network regions) have become attached to the periphery of the ancestral unicellular network, which also has evolved to include new edges. (**C**) Genes and network regions dating to unicellular ancestors (red border) would be preferentially activated in cancer (i.e., have increased expression), while more recently acquired network regions of multicellular ancestors (blue border) would be suppressed.

One of the main evolutionary advances shaping network structure and functionality was the transition from unicellularity to multicellularity (Arenas-Mena, 2017) (Figures 1A and B). Many of the core molecular hallmarks of cancer (Hanahan and Weinberg, 2011) can be interpreted as being due to the dysregulation of genes and cellular processes that appeared during this transition. This is consistent with the observation that neoplasms are not exclusive to high-order organisms but appear across the entire range of multicellular organisms, including metazoans, fungi, plants and algae (Aktipis *et al*, 2015). Thus we hypothesise that the consequences of dysregulation of network regions of different evolutionary ages have fundamentally different effects on tumour formation and progression, dependent on the differing selective pressures that shaped more ancient and more recently evolved network regions.

### Tumours display increased dependence on the activation of ancient network regions

Many phenotypic similarities are evident between cancer cells and unicellular organisms such as bacteria and yeast, including competitive rather than cooperative growth of cells, dependence on fermentation processes used by single-celled organisms (Warburg effect) (Vander Heiden *et al*, 2009), elevated genomic instability reminiscent of mutator phenotypes employed by unicellular organisms under adverse conditions (Yona *et al*, 2012), and stress-induced mutational processes based on ancient DNA repair mechanisms originating in unicellular species (Cisneros *et al*, 2017). These similarities are not coincidental, as cancer-associated genes are enriched in genes conserved with unicellular organisms, suggesting a bias towards activation of more ancient parts of the network during cancer development (Domazet-Loso and Tautz, 2010). This has been described as the atavism hypothesis of cancer, which states cancer occurs when mechanisms employed by differentiated cells to control fundamental cellular processes fail, reactivating primitive transcriptional programs that evolved in the earliest unicellular species (Davies and Lineweaver, 2011; Vincent, 2012) (Figure 1C). This reactivation is thought to be triggered by severe environmental insults similar to those encountered by primitive unicellular organisms, such as nutrient deprivation, hypoxia, reactive oxygen species and low pH. Priority then shifts to the survival of the cell rather than the survival of the organism, promoting malignant transformation as cells proliferate independently.

Results from a recent study suggest selection for an ‘atavistic’ state in cancer (Chen and He, 2016). Here, the functional differences between cell types at the gene expression level were estimated using principal component analysis of gene expression data of 107 different cell types, and tumour tissues and tissues-of-origin of 18 tumour types. They found a consistent progression of all tumour types towards a similar stem-like state, which they argue is not the result of direct selection for pluripotency, but rather selection for unicellular states, due to the simultaneous loss of multicellularity features and the increased prevalence of selection for primitive functions at the cellular level. Additionally, we have previously found even unicellular genes that have been co-opted by predominantly multicellular processes can be activated in cancer (Trigos *et al*, 2017), suggesting a phenomenon of activation of unicellular genes that goes beyond selection for activation of fundamental processes (e.g., cell cycle, metabolism). This view complements the hypothesis that cancer cells access poorly evolved, but defined cellular states (attractors) after disruption of their current state through mutation (Huang *et al*, 2009). This could be interpreted as convergence towards activation of unicellular growth programs by selection for early evolving regions of the network, leading to an ‘atavistic’ phenotype.

Stem cells are another cell type that demonstrates many phenotypic similarities to single-celled organisms, as well as to cancer cells. Comparison of tumour and stem cells reveals the transcriptional state of unicellular programs in tumour cells is distinct from that of stem cells, with primitive stress response mechanisms and metabolic processes diverging in opposite directions in stem and tumour cells (Trigos *et al*, 2017). Therefore, this atavistic cellular state in tumours is distinct to that of the stem cell state, involving upregulation of processes not normally active in stem cells, and provides additional and specific information as to the aetiology of cancer.

In a clinical context, many addictions to specific cellular processes routinely targeted result from a process of atavism. For example, the Warburg effect, a switch to an archaic form of energy production (aerobic glycolysis), has been exploited by PET imaging as a diagnostic tool (Zhu *et al*, 2011). Among the most prescribed cancer drugs are those targeting cellular processes shared with unicellular organisms, including pemetrexed (purine and pyrimidine synthesis), bortezomib (proteasome inhibitor), and paclitaxel and vinblastine (mitotic spindle assembly), alongside many others (Table 1). Furthermore, clinical trials of drugs targeting other fundamental cellular processes, such as the ribosome synthesis inhibitor CX-5461 (Bywater *et al*, 2012) are currently underway (ClinicalTrials.gov Identifier: NCT02719977). Even drugs with indirect effects on unicellular programs are effective, for example, the power of B-Raf inhibitors might be related to the role of B-Raf in controlling glycolysis (Parmenter *et al*, 2014). Such drugs work because of the dependence of tumours on primitive processes, suggesting many forms of oncogene and non-oncogene addiction result from activation of unicellular programs. The newly developed interest in targeting fundamental housekeeping processes by the cancer research community (e.g., Devlin *et al*, 2016) means understanding how the atavistic transformation occurs in cancer holds great promise for the development of new cancer therapeutics.

### Dysregulation of network regions supporting multicellularity in cancer

Metazoan evolution saw the diversification and innovation of new genes, network regions and cellular processes that connected into existing unicellular regulatory networks to provide another layer of control over primitive growth programs (Figures 1A and B). This allowed the emergence of different cell types through the expression of newly evolved proteins with new regulatory, cell–cell signalling and adhesion functions (Ruiz-Trillo and Nedelcu, 2015). This led to the evolution of complex cell communication, cell-to-cell adhesion, differentiation, apoptosis and senescence programs required to support the intercellular communication needed for the development of multicellular organisms, enhancing adaptability through improved ability to adjust their intracellular environment to extracellular cues.

Many of the hallmark properties of cancer can be interpreted as the breaking down of these basic requirements of multicellular tissues via disruption of network regions that formed during the evolution of multicellular species, resulting in increased dependence on primitive unicellular regions (Trigos *et al*, 2017), and implying the core principles of carcinogenesis are linked to the evolutionary history of the molecular network (Figure 1C). Commonly dysregulated molecules and pathways in cancer, such as Wnt, TGF-β, RTKs, Notch/Delta, JAK/STAT, Hedgehog, integrins and cadherins, originally evolved to control the growth programs of unicellular organisms during the transition from unicellularity to multicellularity (Ruiz-Trillo and Nedelcu, 2015). The dysregulation of these network regions leads not only to loss of coordination between cells (Aktipis *et al*, 2015), but also a loss of control over unicellular programs in cancer, whose activation promotes tumour growth.

Recently, evidence of association between the perturbation of genes related to the development of multicellularity with malignancy and metastasis was described (Chen *et al*, 2015). After carrying out serial xenografts in mice of a human breast cell line transfected with HRas, Chen *et al*, found the most highly mutated and consistently downregulated genes in the metastatic samples were enriched in functions related to multicellularity. This is consistent with previous studies showing common cancer-associated genes primarily originated during the early emergence of multicellularity, but not later (Domazet-Loso and Tautz, 2010). These studies support the hypothesis that tumour development and metastasis relies on the functional disruption of genes that sustain multicellularity through control of unicellular programs.

## System-level architecture defined by evolution reinforces cancer development

**Figure 2:**
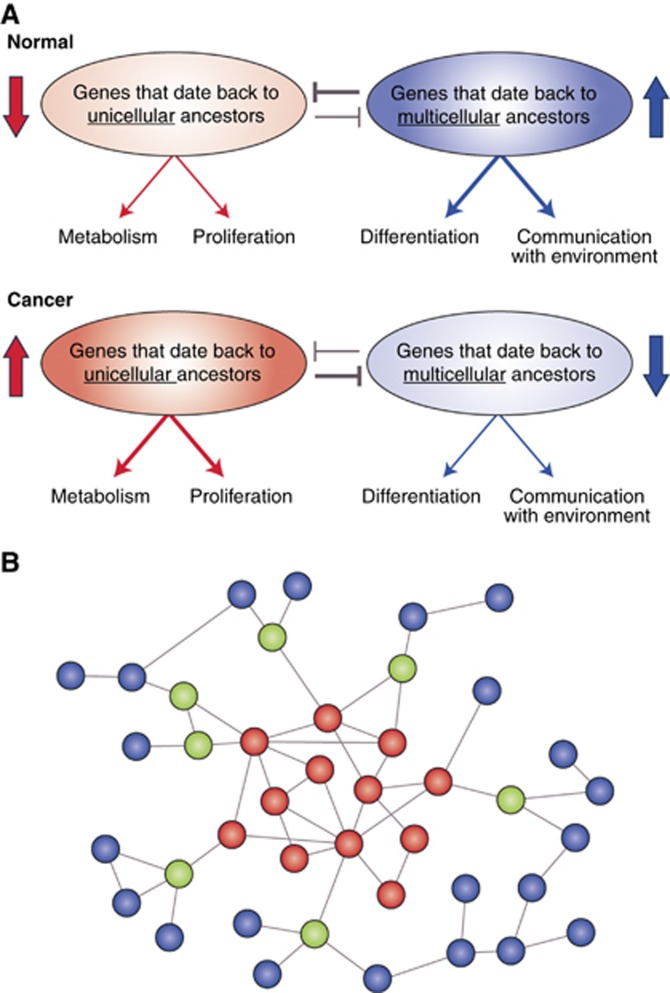
**System-level and gene-level vulnerabilities in cancer defined by evolution.** (**A**) Mutual exclusivity. In normal cells, genes dating to unicellular ancestors that promote primitive cellular functions, such as proliferation and metabolism, are inhibited by more recently acquired upregulated genes promoting differentiation, tissue maintenance and communication with the extracellular environment. This is reversed in cancer, and the activity of more primitive genes suppresses the activity of genes from multicellular ancestors. (**B**) Genes that link unicellular and multicellular network regions (green nodes) are sites of vulnerability, since their disruption would impair communication between unicellular (red nodes) and multicellular (blue nodes) network regions. These vulnerable sites would be enriched in cancer driver genes. An evolutionary analysis of this network can identify such genes, which may not have been apparent from mechanistic laboratory studies.

The differential use of network regions of different age, with network regions that emerged with multicellularity imposing control over ancient unicellular regions, suggests a form of ‘systems-level mutual exclusivity’ whereby activation of processes required for multicellularity would be incompatible with a simultaneous activation of processes that date back to unicellular ancestors (Figures 1C and 2A). This mutual exclusivity in the form of negative correlation of expression of unicellular and multicellular programs has been recently found across multiple normal and tumour types, with an enhancement in tumours, suggesting it is a fundamental characteristic of cells exploited during cancer development (Trigos *et al*, 2017). Importantly, this enhanced mutual exclusivity seems to be characteristic of tumour cells, and is not found in stem cells (Trigos *et al*, 2017).

The well-known relationship between the primitive process of cell cycle progression and the uniquely multicellular process of cell differentiation is probably the best example of apparent mutual exclusivity, where a state of both high replication and cell differentiation cannot simultaneously co-exist. This particular case of mutual exclusivity was identified in the 1970s (Sachs, 1978); it forms the basis for differentiation therapy in leukaemias, and prompts the epithelial–mesenchymal transition that leads to metastasis in solid tumours (Tsai and Yang, 2013). However, this mutual exclusivity between proliferation and differentiation is only one axis of the overall structure of mutually exclusive interactions between cellular processes in tumours, with many more unicellular and multicellular processes with important roles in cancer development exhibiting similar phenomena (Trigos *et al*, 2017). Promoting proliferation, metabolic reprogramming, or other changes while downregulating multicellular processes would support survival and increased malignancy.

Under a general mutual exclusivity framework, the selection for network regions dating back to unicellular ancestors during cancer development would promote loss of multicellular features, which would in turn further increase expression of unicellular genes, creating a feedback loop promoting increasingly malignant states (Figure 2A). During treatment, drugs that effectively promote activation of multicellular processes could impact tumour growth by making a pre-existing atavistic state incompatible with the newly modified, more multicellular state. Examples are drugs that promote the activation of multicellular processes, such as all-trans retinoic acid (ATRA) promoting differentiation, and everolimus promoting senescence, suggesting that the integration of an evolutionary approach helps narrow down similar potential approaches for treatment. Adequate balance and communication between more ancient and more recently evolved network regions is key to avoid entering the feedback cycle that promotes tumourigenesis.

A direct consequence of the mutual exclusivity encoded in the architecture of molecular networks is a limit on the possible evolutionary trajectories available during progression, effectively constraining tumour evolution, much like how synthetic lethal interactions shape tumour progression by limiting what combinations of deleterious mutations are viable. The consequence of this process is perceived as convergent evolution (i.e., the emergence of a common phenotype after the activation of primitive programs inherited from unicellular ancestors) across tumours, which manifests as a common set of hallmarks (Hanahan and Weinberg, 2011) or cellular states (Chen and He, 2016). Thus, major network system-level properties that evolved to overcome past selective pressures, especially during the evolution of multicellularity, have created structures within modern gene regulatory networks that may promote increased malignancy once activated during the initial stages of tumourigenesis.

## Genes at the interface of unicellular and multicellular network regions create vulnerabilities that enable the development of cancer

Considering the fundamental role of communication across network regions of unicellular and multicellular origins, genes at the interfaces between these regions present key points of vulnerability that could lead to tumourigenesis when compromised by somatic mutations or other types of dysregulation, providing targets for broad-spectrum therapeutics. The existence of specific regions in the cellular network of human cells with intrinsic vulnerability to cancer has previously been reported. For example, genes with mutations causally implicated in oncogenesis cluster in specific regions of the molecular network, especially central but fragile regions of the network that link multiple network regions (intermodular hubs) (Jonsson and Bates, 2006). These network regions of vulnerability correspond to genes modulating the complexity needed for multicellularity, and would be composed of oncogenes and tumour suppressors, which mostly emerged at the onset of multicellularity (Domazet-Loso and Tautz, 2010) (Figure 2B). Their position as highly linked intermodular hubs (Jonsson and Bates, 2006) supports their role as integrators of unicellular and multicellular network regions.

These vulnerabilities can be considered a necessary tradeoff to achieve the advantages conferred by increased organismal complexity (Aktipis *et al*, 2013). While their location in the network is a result of past evolutionary processes, this configuration poses a risk to the loss of a multicellular phenotype after disruption of just a few key genes. Damage to these genes will substantially alter communication and balance between pathways required to maintain a multicellular state and those regulating basic cellular survival mechanisms, thus uncoupling the cell from its environment. This uncoupling is reflected as proliferation independent of growth factors and evasion of contact inhibition in cancer (Hanahan and Weinberg, 2011).

An important example of such a point of vulnerability is the master regulator p53, which acts as a regulatory hub for a diverse set of processes, but has little functional overlap with other molecules, representing a high risk to the maintenance of structural integrity and stability of the network. Importantly, the orthology of this gene extends back to primitive multicellular organisms (Belyi *et al*, 2010), marking it as a potential master regulator of the interaction between unicellular and multicellular network regions (Figure 2B, green nodes), adding a new dimension to the relevance of this gene to cancer. Other examples of genes regulating the communication between unicellular and multicellular network regions have been found to be potential clinical targets in cancers with heterogeneous activation of oncogenic pathways. Regulators of translation initiation, such as eIF4E, date back to unicellular ancestors and are responsible for the translation of genes involved in cell survival and replication (Mamane *et al*, 2004). The evolution of complexity resulted in multicellularity-related pathways such as Akt, Ras and MAPK converging on eIF4E, further regulating angiogenesis, autocrine growth stimulation and communication with extracellular cues (Mamane *et al*, 2004)-all stimulators of tumour development. This creates a vulnerable network topology that results in a consistent convergence to the upregulation and increased phosphorylation of eIF4E directed by a broad set of oncogenes (Bhat *et al*, 2015), reinforcing the importance of mediators of communication between genes of unicellular and multicellular origin in cancer aetiology.

## Association of drug resistance mechanisms with robust network regions defined by evolution helps explain the high resilience of cancer to treatment

**Figure 3:**
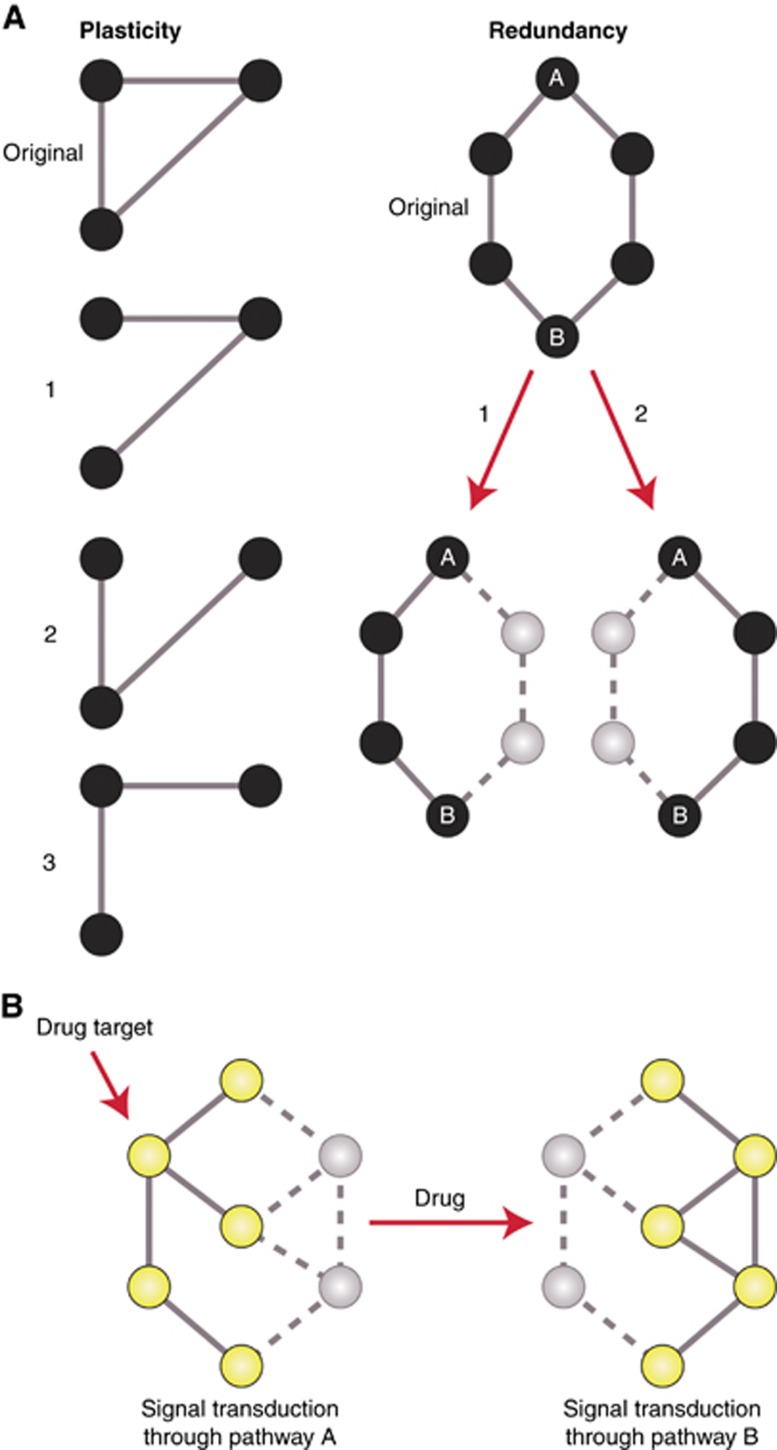
**Robustness of networks and its effect on the development of drug resistance in cancer.** (**A**) (Left) Representation of network plasticity. High connectivity of nodes allows communication to be maintained even after removal of edges (states 1–3). (Right) Representation of network redundancy. The degree of communication between nodes A and B is not affected even after removal of a direct path, given the existence of redundant, equivalent paths of communication. Plasticity and redundancy define the robustness of a network. (**B**) Robustness allows the action of drugs to be overcome. Initially, signal transduction occurred from the top to the bottom node through the yellow, upregulated nodes and solid edges, with a parallel, inactivated pathway having little contribution (grey nodes, solid edges). After drug inhibition of the active pathway, the parallel pathway can be activated and restore signalling, leading to drug resistance. Given the high robustness of unicellular network regions, the development of resistance and the ability to withstand genetic changes would preferably occur in these regions.

Owing to their essentiality for the survival of cells, evolution has maintained the homeostasis of the most fundamental, primitive cellular processes by enforcing redundancy and plasticity. Plasticity refers to the ability of a network to rewire (permanently or transiently) without fatal damage, and is a commonly adopted concept in the context of cancer stem cells, epithelial–mesenchymal transitions, and drug resistance. Network plasticity, together with redundancy, avoids the loss of communication after damage of linking regions (Figure 3A), and defines the robustness of a network (Kitano, 2004); that is, its ability to appropriately respond to stimuli while adapting to intrinsic and extrinsic selective pressures and alterations.

Robustness is also highly tied to the underlying evolutionary structure of the network due to its association with the distribution of the number of interactions (degree) of genes (Kim *et al*, 2014), which in turn reflect their evolutionary history (Chen *et al*, 2014). Therefore, increased reliance on unicellular network regions, which are highly robust, as part of a process of atavism could benefit cancers by providing greater resilience to the selective pressures encountered during its life history (Figure 3B), such as hostile microenvironments, stress from increased DNA damage and chemotherapy. This can also help explain why convergence to similar cellular destinations that favour proliferation is observed across tumours (Chen and He, 2016).

Under a framework of co-selection of proliferation programs and robustness, it is not surprising that many common resistance mechanisms are confined primarily to unicellular network regions. Examples include the increased activity of DNA repair pathways in response to cisplatin, and the action of membrane pumps that extrude common cancer therapeutic drugs, specifically those belonging to the superfamily of ATP-binding cassette (ABC) proteins, whose targeting has been proposed as a therapeutic strategy derived from their evolutionary properties (Lineweaver *et al*, 2014). Both mechanisms are powered by ancient processes that date back to unicellular ancestors: DNA repair mechanisms originally evolved to withstand UV radiation, while ABC proteins have been found across the tree of life, including prokaryotes and eukaryotes (Vasiliou *et al*, 2009). This suggests the development of resistance may be driven by chemotherapy-induced general reliance on robust unicellular programs.

A more detailed picture of how robustness modulates resistance comes from a study where multiple myeloma cells were treated with doxorubicin, an inhibitor of DNA replication, until resistant cells emerged (Wu *et al*, 2015). Comparison of gene expression levels and somatic mutational burden between resistant and wild-type cells revealed that genes with a four-fold or higher change in expression in resistant cells tended to be more highly conserved, but had fewer mutations. This could be interpreted as activation of selected ancient network regions during the development of doxorubicin resistance, with the lack of mutations indicating preferential preservation of their functional integrity due to their essentiality in surviving doxorubicin treatment. This suggests that robustness established by the evolutionary history of the network plays a role in defining the type of perturbations different network regions are able to withstand in cancer during the development of drug resistance, and probably also during progression.

Selection for activation of robust network regions of specific evolutionary age during carcinogenesis has important implications for how and why cancer cells are so adept at developing drug resistance, and can withstand high levels of genomic instability and hostile microenvironments. The structure of the network defined by evolution limits which alterations to the connections between genes and network regions can be tolerated, affecting the possible trajectories for cancer to take during the development of resistance.

## Designing therapeutic strategies linking evolution with network medicine

The tight association between the mechanisms of tumourigenesis and the evolutionary history of the network makes integrating these two approaches attractive. Here we propose therapeutic strategies where the evolutionary history of the network provides a meaningful guide to define network regions and genes to target, and to develop system-level therapies of broad efficacy.

### Strategy 1: Targeting cellular processes of specific evolutionary age: unicellular processes

Although selectively targeting fundamental cellular processes poses multiple challenges given their role in the survival of normal cells, it has been shown specific basal processes can be selectively targeted in cancer cells. For example, RNA polymerase I (PolI) transcription strategies that inhibit ribosome synthesis are selective to malignant B cells, because of different thresholds in normal and cancer cells for the drugs to elicit a response as a result of network rewiring (Bywater *et al*, 2012; Hannan *et al*, 2013). This and similar approaches are currently being tested in clinical trials, and many commonly used drugs in the clinic target unicellular programs (Table 1). Thus, studies that associate cancer development with the evolutionary history of genes, molecular networks and genomic approaches provide an alternative, potentially more direct method of identifying regions upon which tumours have increased dependence or addiction. Given emerging evidence of reliance on unicellular genes as a fundamental property of cancer, identification of reactivated unicellular programs and the main players behind this activation (genes, pathways, network modules) would help prioritise therapeutic targets with greater specificity to cancer cells.

Recently, a framework of robustness and redundancy has proven useful in the discovery of targets to overcome chemotherapy resistance (Azevedo and Moreira-Filho, 2015) and in identifying drug combinations for breast cancer (Jaeger *et al*, 2016). However, the further incorporation of an evolutionary context will increase the predictive power of treatment outcomes and guide the design of targeted and combination therapies by narrowing down regions of different degrees of robustness. We propose that while highly conserved network regions are highly robust and a source of drug resistance mechanisms, therapies targeting regions that evolved in multicellular organisms or that are less conserved with unicellular species would be more likely to be effective. Furthermore, investigating how the shift to an atavistic state allows cancer cells to take advantage of the resilience encoded in unicellular components of their cellular network, and the general principles governing this robustness, would highlight regions of low-plasticity and redundancy that would make attractive drug targets.

### Strategy 2: Exploiting network configurations: mutual exclusivity between unicellular and multicellular processes

Constraints imposed by the mutual exclusivity between unicellular and multicellular network regions can be exploited therapeutically. Owing to the incompatibility of a simultaneous activation of unicellular and multicellular processes, applying environmental stressors that promote the activation of multicellular programs would push cells towards the compensatory inactivation of unicellular processes. The intrinsic reliance of cancer cells on these unicellular processes would make these cells lose their fitness advantage, rendering them unviable.

Stimulation of multicellular programs can be achieved directly by promoting the activation of mutated hallmark genes controlling multicellular processes (e.g., by the activation of mutant p53 using small molecules; Bykov and Wiman, 2014). Another approach would be to indirectly activate multicellular programs by inhibiting unicellular ones. For example, it has been shown that inhibiting fundamental processes such as ribosome biogenesis leads to the activation of p53 (e.g., Bywater *et al*, 2012), inhibition of glycolysis (unicellular program) increases sensitivity of cancer cells to the induction of apoptosis (multicellular program) (Meynet *et al*, 2012), and cancers accumulating mutant p53 protein can be targeted by inhibiting an antiporter involved in redox modules that date back to unicellular ancestors (Liu *et al*, 2017).

An advantage to this approach is that targeting multicellular programs in itself would be effective in avoiding resistance, given that their short evolutionary history and lack of strong past selective pressures would mean fewer redundant mechanisms and less robustness. Multicellular processes might be damaged in tumours to the point where they are unable to be activated, even after induction by drugs, leading to death of cancer cells but not normal, healthy ones. Therefore, a complementary therapeutic strategy would be to apply stressors that require activation of multicellular processes that have been irreversibly damaged in cancer cells.

Establishing the extent of mutual exclusivity between network regions or cellular processes of different evolutionary age would help pinpoint the most promising targets. The approaches that are most promising for this strategy would be those incorporating the entire wiring of cellular networks, by focusing on steering network traffic to desired states by specific ordering and timing of multiple drugs (Russell and Aloy, 2008). Development and implementation of these approaches would allow a reprogramming of the balance of unicellular and multicellular network regions, either by shifting cells back to their original configuration, or reducing their fitness and allowing them to be removed by selection.

### Strategy 3: Deriving targetable points of vulnerability at the interface of unicellular and multicellular regions

Another strategy would be to directly target communication between unicellular and multicellular processes by targeting genes at the interfaces between these network regions. An inherent property of biological systems is fragility at specific sites that leads to system failure if perturbed (Carlson and Doyle, 2002), much like the genes that emerged early in multicellularity as necessary tradeoffs for the development of complexity (Aktipis *et al*, 2013). Drugs that target these regions would lead to a fractioning of the network along evolutionary lines, possibly showing pan-cancer effectiveness.

Points of vulnerability can be derived by network-based methods, to locate hub genes, intermodular hubs or key edges (interactions between proteins) connecting network regions of different age. Dating of network regions or genes can be achieved by systematically determining the evolutionary ages of genes and cellular processes, using algorithms that compare the increasing diversity of available genomes to detect sequence similarity across species (e.g., OrthoMCL; Li *et al*, 2003) or by comparison of networks between species of differing levels of complexity. The development of methods that integrate basic phenotypic features of cells, such as genome-wide expression levels, with evolutionary information would further allow the discovery of trends.

Another approach to deriving these points of vulnerability is through deeper understanding of the key genes and processes required for multicellularity. Studies of simple multicellular organisms and their close unicellular relatives are uncovering key regulators of the transition from unicellularity to multicellularity. Investigating whether the human orthologues of genes governing the balance between colonial and unicellular states are mutated or dysregulated in tumours would be one way to effectively pare down the long gene lists emerging from tumour sequencing studies and functional genomics screens of cancer cell lines, and reveal promising candidate genes that could be targeted with high specificity.

Of course, as with any cancer drug, new therapies targeting such points of vulnerability could face challenges in achieving high tumour-cell specificity and effective therapeutic ratio. There are many practical limitations involved in restoring pathways inactivated by loss-of-function mutations as well. However, activating changes such as gain-of-function mutations, epigenetic alterations, changes in gene expression or copy-number variations that alter or create new links between unicellular and multicellular processes would be more amenable to pharmacological intervention (Trigos *et al*, 2017). Additionally, a network approach could be used in response to loss-of-function mutations, searching a gene’s downstream or upstream neighbours in the network or genes in parallel pathways for those that could be targeted instead of the gene carrying the loss-of-function mutation.

## Conclusion

Cancer development is greatly influenced by how the structure of human molecular networks was shaped by past evolutionary processes, but the potential benefits of incorporating evolutionary information are rarely appreciated in genomic studies of cancer. In particular, the transition from unicellularity to multicellularity left vulnerabilities in the network structure that are particularly relevant to cancer. Many properties of multicellular organisms are dysregulated or lost in this disease and genes dating back to unicellular ancestors are either specifically activated during transformation and/or required for maintenance of cancer phenotype. Incorporating the evolutionary origins of genes in systems-level analyses of cancer will provide a better understanding of how alterations to the sequence or regulation of genes in cancer may affect molecular networks, explain why specific alterations are preferred in cancer cells, and indicate which phenotypic properties are under selection during progression. Thus, studies investigating the loss of multicellular features and concurrent appearance of more unicellular-like phenotypes during progression and metastasis would provide predictive power for analysis of the system-level changes and key pathways driving later stages of tumour development. Furthermore, investigating whether the development of resistance is confined to highly robust network regions adaptable to change, and having a better understanding of which alterations at the genetic, transcriptional and protein levels are and are not tolerated would help narrow down the network regions and pathways involved in resistance mechanisms. This can be incorporated in the design of therapies from a network medicine or system-level approach that *a priori* takes into account the development of resistance. By incorporating the evolutionary analysis of genes and their interactions in the study of cancer, we will gain a broader picture of system-level properties, vulnerabilities and network rewiring that occurs in cancer, and be in a better position to understand common principles behind multiple tumour types and tackle inter-patient tumour heterogeneity. This will enhance and accelerate development of treatment strategies that exploit network configurations with the potential for broad-spectrum cancer therapies.

## Figures and Tables

**Table 1:** Cancer therapeutics targeting highly conserved unicellular processes

**Drug**	**Mechanism of action**	**Targeted cancer type**	**Reference/reviewed in**
Pemetrexed	Purine and pyrimidine synthesis	Lung cancer	Hanna *et al* (2004)
Bortezomib	Proteasome	Myeloma and lymphoma	Piperdi *et al* (2011), Richardson *et al* (2003)
Vincristine, vinblastine, vinorelbine	Suppression or depolymerisation of microtubules, destruction of mitotic spindles	Broad spectrum	Jordan and Wilson (2004)
Paclitaxel	Microtubule-stabilisation, inducing mitotic arrest	Ovarian, breast, lung, gastroesophageal, endometrial, cervical, prostate, and head and neck cancers, sarcoma, lymphoma, and leukaemia	Weaver (2014)
Camptothecin	Targets DNA topoisomerase I	Broad spectrum	Liu *et al* (2000)
